# The prevalence of *Listeria* species in different food items of animal and plant origin in Ethiopia: a systematic review and meta-analysis

**DOI:** 10.1186/s40001-021-00532-8

**Published:** 2021-06-24

**Authors:** Kuma Diriba, Ephrem Awulachew, Kuma Diribsa

**Affiliations:** 1 Department of Medical Laboratory Sciences, Health Science and Medical College, Dilla University, Dilla, Ethiopia; 2 Department of Biology, Educational College, Dilla University, Dilla, Ethiopia

**Keywords:** Listeria, Prevalence, Milk products, Animal product, Ethiopia

## Abstract

**Background:**

Listeriosis is one of the important emerging zoonotic disease affecting human health following the consumption of contaminated food of animal origin. It results in serious clinical complications in humans with a high case facility rate. Therefore, this systematic review and meta-analysis aimed to estimate the pooled prevalence of *Listeria* species in Ethiopia.

**Methods:**

A systematic search was conducted on PubMed, Web of Science, EMBASE, Google Scholar and the Cochrane Library. All identified observational studies reporting the prevalence of *Listeria* species in different food items of animal and plant origin in Ethiopia were included. Three authors independently extracted data and analyzed them using STATA Version 13 statistical software. A random effects model was computed to estimate the pooled prevalence of *Listeria* species in Ethiopia.

**Results:**

After reviewing 122 studies, five studies fulfilled the inclusion criteria were included in the meta-analysis. The findings from the five studies revealed that the pooled prevalence of *Listeria* species in different food items of animal and plant origin in Ethiopia was 27% (95% CI 25, 29). The highest prevalence of *Listeria* species was reported in beef meat followed by ice cream with prevalence rates of 62% (95% CI 50, 75) and 43% (95% CI 33, 53), respectively.

**Conclusion:**

The presence of *Listeria* species in different food items of animal and plant origin in Ethiopia is an indicator of the presence of public health hazards to the consumer, particularly to the high-risk groups. Hence, awareness creation on food safety and implementation of regulations is strongly recommended.

## Background

Listeriosis is an important emerging zoonotic disease affecting human health following the consumption of contaminated foods of animal origin [1]. Among the different species of the genus *Listeria*, *L. monocytogenes* is the causative agent of listeriosis [2]. *L. monocytogenes* can persist for long periods in the environment or as an asymptomatic infection in adult animals and birds due to its psychrophilic nature, it can grow within a wide range of temperatures (− 1.5 to 50 ºC) [3]. *L. monocytogenes* can persist for long periods in the environment and can easily contaminate agricultural products and ultimately livestock products [4].

*Listeria monocytogenes* is an important cause of diseases in both animals and humans. In the vast majority of human cases, infection is the result of consumption of contaminated food. *L. monocytogenes* is a food-borne pathogen, and its incidence and growth in food, contribute to outbreaks of listeriosis [5]. Reports have indicated that *Listeria* spp. including *L. monocytogenes* is most frequently prevalent in the milk-processing environment including steps, drains and floors [2]. In addition, different studies [6–8] reported that a higher prevalence of *Listeria* species was also found in meat and meat product foods, and ice cream samples.

The occurrence of listeriosis among humans has received increasing attention as epidemic listeriosis has been recognized and reported in immunosuppressed populations [9]. This bacterium principally causes intrauterine infection, meningitis and septicemia. Listeriosis in pregnancy may be asymptomatic or manifest as severe systemic infection in the unborn or newly delivered infants. The microorganism causes fatal infections such as encephalitis, sepsis and meningitis in immune deficient patients and abortion in pregnant women [10]. The mortality rate is reported to be 20–30% [11, 12].

Currently, there is inadequate information regarding the prevalence of *Listeria* species in Ethiopia. Therefore, the present systematic review was undertaken to determine the pooled prevalence of *Listeria* species using a published article in Ethiopia.

## Methods

### Study design

A systematic review and meta-analysis were conducted to estimate the magnitude of *Listeria* species in different food items of animal and plant origin in Ethiopia following the methodological framework of systematic review and by checking the following five steps: step 1: framing questions for a review, step 2: identifying relevant work, step 3: assessing the quality of studies, step 4: summarizing the evidence, and step 5: interpreting the findings [13].

### Search strategies

All relevant articles were searched using the following databases: PubMed, Web Science, Embase, Google Scholar, Cochrane Library and Science Direct according to the Preferred Reporting Items for Systematic Reviews and Meta-analysis (PRISMA) [14]. All searches were limited to articles written in English given that such language restriction does not alter the outcome of the systematic reviews and meta-analysis [15]. The gray literature of observational studies was searched through the review of reference lists and input of content experts. The literature search was conducted from January 2009 to February 2015. All papers published until the end of 2015 and fulfilling inclusion criteria were considered. The search used the following keywords “listeria”, “prevalence”, “milk products”, “animal product”, and “Ethiopia “. The search terms were used separately and in combination using Boolean operators such as “OR” or “AND”.

### Eligibility criteria

Studies conducted only in Ethiopia and involving only humans were included in this study. Publication condition: only published articles were included. Study design: all observational study designs reporting the prevalence of Listeria species in animal and plant product were eligible for this review. Language: only articles reported in the English language were considered. Exclusion criteria: articles, that were not fully accessible, after email contact with the primary authors and duplicate publications of the same study, were excluded.

### Assessment of study quality

Studies selected for inclusion were assessed for methodological quality by all authors independently using the standard critical appraisal instruments of the Joanna Briggs Institute Meta-analysis of Statistics Assessment for Review Instrument (JBI-MAStARI) [16]. Disagreements were resolved by consensus.

### Outcome measure

The primary outcome variable of this study was the prevalence of *Listeria* species, while the secondary outcome was its comparison in different types of food, including cheese, raw beef, raw milk, fish meat, raw meat, ice cream, and cream cake.

### Data extraction

Data were extracted using a standardized data extraction format, adapted from the Joanna Briggs Institute (JBI), by three authors (Kuma Diriba, Ephrem Awulachew and Kumsa Diriba) independently extracting all necessary data. Then, the extracted data were merged for systematic analysis. Any disagreements during the data extraction were resolved through discussion and consensus. The main outcomes extracted from the study were: primary author, publication year, study method, study area, sample size and cases. Data on associated risk factors were also extracted by the authors.

### Statistical analysis

Following data extraction, systematic review and meta-analysis were carried out using R software version 3.6.1 and STATA statistical software (version 13) with user contributed commands for meta-analyses: metaprop, metan, metainf, metabias, and metareg [17]. The effect sizes and SEs of the studies were pooled using a random-effects model to calculate the pooled prevalence of *Listeria* species in different food items of animal and plant origin in Ethiopia. A meta-analysis was also planned to assess the association *Listeria* species in different food items of animal and plant origin collected during the study period.

The standard error for each original study was calculated using the binomial distribution formula. Evidence for statistical heterogeneity among reported prevalence was using the Cochrane *Q*-test and *I*^2^ statics [18]. The pooled proportion was estimated using the back-transform of the weighted mean of the transformed proportions for both the fixed-effects model and the random-effects model [19]. A significance level of *P* < 0.10 and *I*^2^ > 50% was interpreted as evidence of heterogeneity [20]. A potential source of heterogeneity was investigated by subgroup analysis and meta-regression analysis [21]. Where statistical pooling was not possible, the findings were presented in a narrative form including tables and figures to aid in data presentation where appropriate.

### Risk of bias

Three authors (KD, EA and KD) independently assessed the risk of bias for each original study using the 10 criteria tool of Hoy 2012 which addresses internal and external validity [55]. The tool mainly included [1] representation of the population, [2] sampling frame, [3] methods of participants’ selection, [4] non-response bias, [5] data collection directly from subjects, [6] acceptability of case definition, [7] reliability and validity of study tools, [8] mode of data collection, [9] length of prevalence period, and [10] appropriateness of numerator and denominator. Each item was classified as either low or high risk of bias. Finally, the overall score of risk of bias was then categorized into low [2], moderate [3, 4], and high (> 5) out of 10 and almost all of the original study fall under low risk of bias.

Sensitivity analyses were conducted to weigh up the relative influence of each individual study on the pooled effect size using a user-written function, metainf. The presence of publication bias was assessed informally by visual inspection of funnel plots [22]. Point prevalence, as well as 95% confidence intervals, was presented in the forest plot format.

## Results

### Study selection

**Fig. 1:**
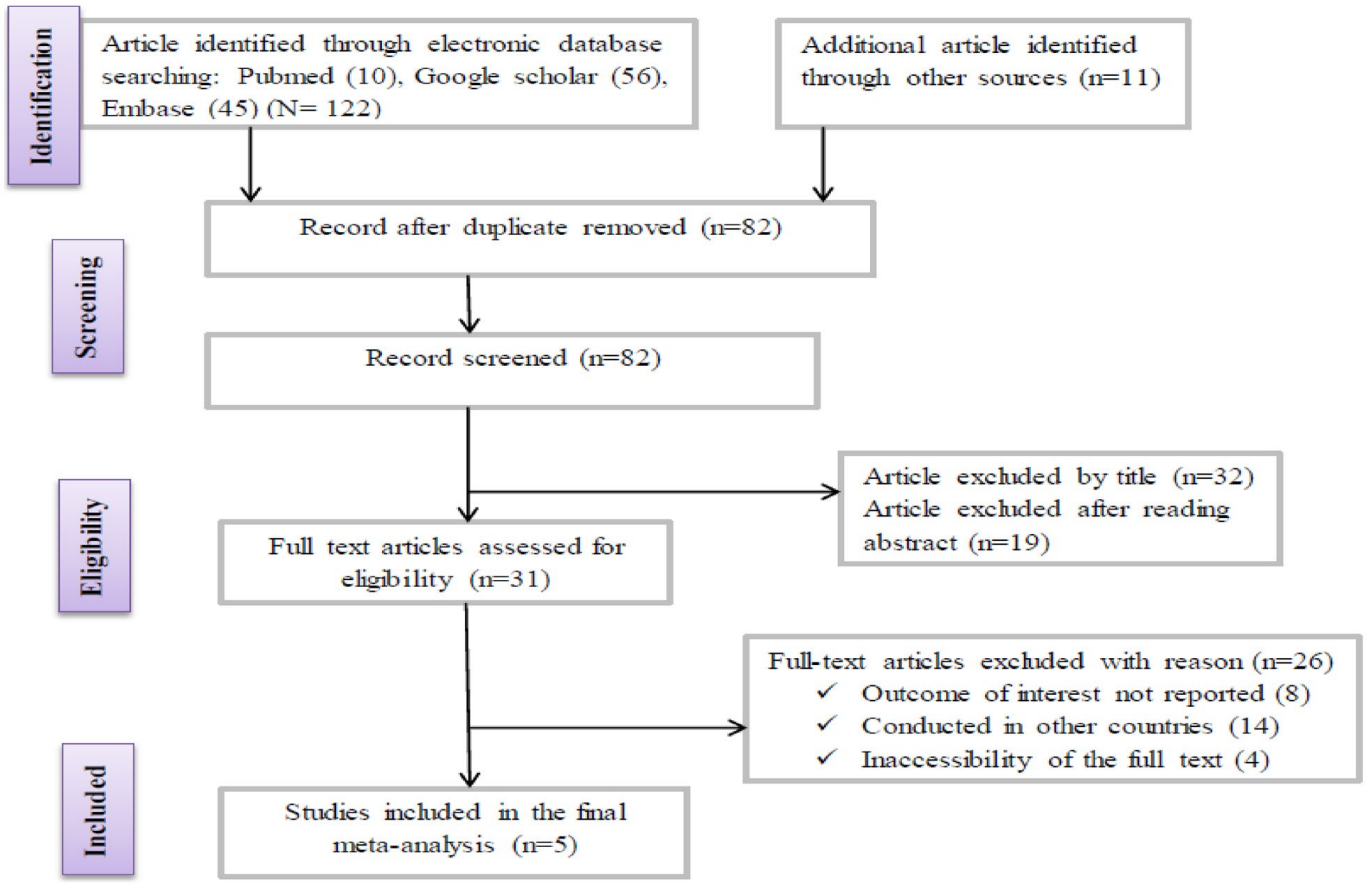
Flow chart of study selection for systematic review and meta-analysis of the prevalence of Listeria species in different food items of animal and plant origin

Data base search identified a total of 122 articles reporting prevalence of *Listeria* species in different food items of animal and plant origin. From these initial articles, 40 articles were excluded due to duplication. From the remaining 82 articles, 51 articles were excluded after review of their titles and abstracts confirmed non-relevance to this review, 31 full-text articles were assessed with respect to their eligibility for inclusion, which resulted in the further exclusion of 26 articles primarily due to the study done in other countries [23–49], and 5 studies were included in the final systematic review and meta-analysis (Fig. 1).

### Description of included studies

In this review, the five papers published between, 2009 and 2015 and reported prevalence of *Listeria* species in different food items of animal and plant origins were included. In this systematic review and meta-analysis, 2169 study participants were included to assess the pooled prevalence of *Listeria* species among food and animals products. Sample size of the included studies was ranged from 240 to 711. The prevalence of *Listeria* species in different areas reported in this meta-analysis was near to each other with the lowest prevalence (25%) reported in in Gondar town, Amhara [50] and the highest prevalence (28%) in Addis Ababa [51, 52]. Three of the studies were from Addis Ababa [51–53], and two from Amhara [6, 50] (Table 1).

**Table 1:** Description of five studies included in the meta-analysis of the prevalence of *Listeria* species in different food items of animal and plant origin in Ethiopia, 2020

First authors	Publicatio*n* year	Study method	Study area	Region	Sample size	Cases	Prevalence with 95% CI
Mengesha et al. [6]	2009	Cross-sectional	Gondar town	Amhara	711	189	27 (23, 30)
Seyoum et al. [51]	2015	Cross-sectional	Addis Ababa	Addis Ababa	443	126	28 (24, 33)
Derra et al. [52]	2013	Cross-sectional	Addis Ababa	Addis Ababa	240	66	28 (22, 34)
Kebede et al. [53]	2010	Cross-sectional	Addis Ababa	Addis Ababa	391	102	26 (22, 31)
Garedew et al. [54]	2015	Cross-sectional	Gondar town	Amhara	384	96	25 (21, 30)

### Risk of bias

The risk of bias tool [55] was used to assess the risk of bias for the included studies and almost greater than 80% of the studies had low risk of bias. The sample selection and temperature during transport and the amount of any individual sample tested were specified in some of the studies. *Listeria* specific enrichment media, biochemical test and supplement were used in majority of the studies. *Listeria* was incubated at 30 °C for 24–48 h in most of the studies.

### The magnitude of *Listeria* species in Ethiopia

**Fig. 2:**
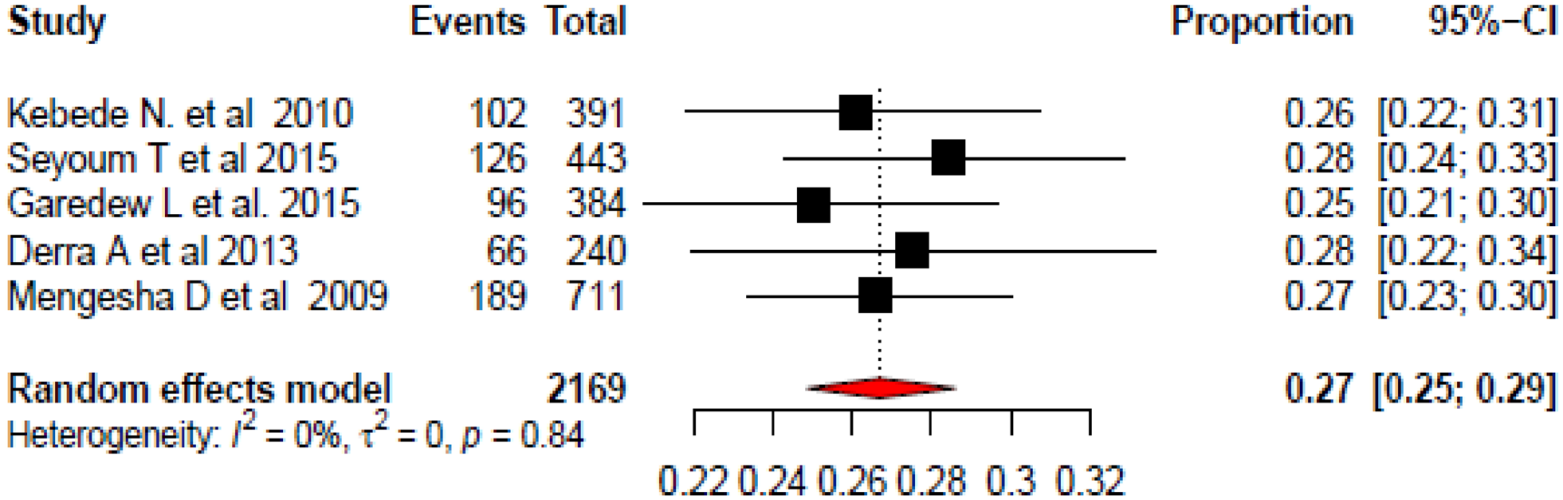
Forest plot of the pooled prevalence of Listeria species in different food items of animal and plant origin in Ethiopia, 2020

The pooled prevalence of *Listeria* species in different food items of animal and plant origin in Ethiopia was 27% (95% CI 25, 29). The heterogeneity observed across the included studies was zero (*I*^2^ = 0, *p* = 0.84). From this meta-analysis, the prevalence of each study was nearer to each other falling within the range of 25% and 28% (Fig. 2).

Based on our finding, there are about seven species of listeria commonly reported throughout the country from which *L. ivanovii* was the predominant listeria isolate followed by *L. welshimeri* with prevalence of 149 (29.7%) and 147 (29.3%), respectively. The highest prevalence of *L. monocytogenes* was reported in study conducted in Gondar town [6] with prevalence of 32.7%, while *L. seeligeri* was the least listeria isolate with prevalence of 14 (2.8%) (Table 2).

**Table 2:** Prevalence of listeria species in different study area found in Ethiopia, 2020

Author	Study area	*L. monocytogenes*	*L. ivanovii*	*L. innocua*	*L. seeligeri*	*L. welshimeri*	*L. grayi*	*L. murrayi*
Mengesha et al. [6]	Gondar town	34 (32.7%)	126 (84.6%)	4 (2.7%)	5 (35.7%)	13 (37.1%)	1 (4%)	6(22.2%)
Seyoum et al. [51]	Addis Ababa	25 (24%)	19 (12.8%)	33 (22.4%)	12 (85.7%)	6 (17.1%)	19 (76%)	12 (44.4%)
Kebede et al. [53]	Jimma town	21 (20.2%)	2 (1.3%)	62 (42.2%)	4 (28.6%)	7 (20%)	3 (12%)	3 (11.1%)
Garedew et al. [54]	Gondar town	24 (23.1%)	2 (1.3%)	48 (32.7%)	5 (35.7%)	9 (25.7%)	2 (8%)	6 (22.2%)
Pooled prevalence of each species		104 (20.8%)	149 (29.7%)	147 (29.3%)	14 (2.8%)	35 (7.0%)	25 (5.0%)	27 (5.4%)

### Prevalence of *Listeria* species in different food items of animal and plant origin in different study area in Ethiopia

In this systematic review and meta-analysis, we tried to assess the distribution of *Listeria* species in different food items of animal and plant origin. The highest and the lowest contamination of *Listeria* species was reported from Gondar town with highest prevalence 28% [54] and the lowest prevalence 0.0% [6] which may be due to study period. The highest prevalence of *Listeria* species in beef meat was reported in Addis Ababa [52] with prevalence of 62%, but the lowest prevalence was reported in Gondar town [54] with a rate of 24%. Higher prevalence of *Listeria* species in cream (43%) and in egg (32%) was reported in Amhara region [6] and Addis Ababa [51], respectively. However, the lowest prevalence of *Listeria* species in cream (23%) and egg (16%) was reported in Addis Abba [52] and Gondar town [6], respectively (Table 3).

**Table 3:** Magnitude of Listeria species in different food items of animal and plant origin in Ethiopia, 2019

Author	Year	Region	Study area	Cheese *P* (95% CI)	Beef meat *P* (95% CI)	Raw milk *P* (95% CI)	Cream *P* (95% CI)	Egg *P* (95% CI)
Mengesha et al. [6]	2009	Amhara	Gondar town	0 (0, 7)	48 (38, 57)	0 (0, 7)	43 (33, 53)	16 (11, 22)
Seyoum et al. [51]	2015	Addis Ababa	Addis Ababa	21 (17, 26)	ND	21 (17, 26)	ND	32 (24, 42)
Derra et al. [52]	2013	Addis Ababa	Addis Ababa	10 (3, 24)	62 (50, 75)	10 (3, 24)	23 (13, 34	ND
Kebede et al. [53]	2014	Oromia	Jimma Town	22 (14, 31	51 (40, 63)	22 (14, 31)	ND	ND
Garedew et al. [54]	2015	Amhara	Gondar town	28 (16, 42)	24 (9, 45)	28 (16, 42)	23 (15, 34)	ND
Pooled prevalence				10 (6, 16)	48 37, 60)	13 (5, 29)	29 (20, 41)	23 (13, 36)

*ND* not done in original article; *P* (95% CI) prevalence with 95% confidence interval

### The distribution of *Listeria* species in different food items of animal and plant origin

Regarding their distribution in different food item, *L. innocua* was the leading isolate in cheese with prevalence of 52.9%, followed by *L.monocytogenes *(17.8%) and* L. murrayi *(11.8%).* L. innocua* was the predominant isolated *Listeria* in raw beef, cream and egg with prevalence of 61.1%, 48.6% and 72%, respectively, while *L. grayi was* the least isolate in raw beef and cream with prevalence ranged from 0.0 to 1.4% and *L. murrayi* was the least isolate in egg with prevalence of 0.0% (Table 4).

**Table 4:** Distribution of *Listeria* species in different food items of animal and plant origin (chees, raw beaf, raw milk, cream, egg) in Ethiopia, 2020

*Listeria* species isolated	Cheese	Raw beef	Raw milk	Cream	Egg	Total
*L. monocytogenes*	9 (17.6%)	12 (11.1%)	22 (25.6%)	22 (29.7%)	8 (16.0%)	73 (19.8%)
*L. ivanovii*	2 (3.9%)	12 (11.1%)	12 (14.0%)	8 (10.8%)	1 (2.0%)	35 (9.5%)
*L. innocua*	27 (52.9%)	66 (61.1%)	11 (12.8%)	36 (48.6%)	36 (72.0%)	176 (47.7%)
*L. seeligeri*	3 (5.9%)	3 (2.8%)	12 (14.0%)	1 (1.4%)	1 (2.0%)	20 (5.4%)
*L. welshimeri*	2 (3.9%)	14 (13.0%)	4 (4.7%)	2 (2.7%)	2 (4.0%)	24 (6.5%)
*L. grayi*	2 (3.9%)	0 (0.0%)	18 (20.9%)	1 (1.4%)	2 (4.0%)	23 (6.2%)
*L. murrayi*	6 (11.8%)	1 (0.9%)	7 (8.1%)	4 (5.4%)	0 (0.0%)	18 (4.9%)
Total	51 (13.8%)	108 (29.3%)	86 (23.3%)	74 (20.1%)	50 (13.6%)	369 (100%)

## Discussion

Data on *Listeria* species in different food items of animal and plant origin in Ethiopia are limited and are not currently available in aggregate form. We conducted a systematic review and meta-analysis to provide the pooled prevalence of Listeria species in different food items of animal and plant origin. *Listeria* species are a leading cause of bacterial-derived foodborne disease worldwide with an estimated 400 million cases per year [56]. The information from this study might be used by the policy makers in the prevention and control of the infection.

In the present study, the pooled prevalence of *Listeria* species in different food items of animal and plant origin obtained from this study was 27% that indicated significant public health hazard associated with consumption of contaminated foods of animal and plant origin. This indicates, in part, increased attention to the issues of microbial food safety in this region. The result of this meta-analysis is in line with study conducted in Malaysia [57], Chile [58], Uganda [59] and Botswana [60]. However, our result is higher than the study conducted in Ghana [61] and elsewhere [51] and lower than study conducted in Turkey [62] and Norway [63]. The possible explanation for the above variation may be methodological difference, differences in food items composition or hygienic status of different food items of animal and plant origin.

In the current study, both food items of animal and plant origin showed a significant level of contamination with *Listeria* species. In this study, cheese, raw beef, raw milk and liquid whole egg samples were collected and analyzed for the presence of *L. monocytogenes* and other *Listeria* species. Among the seven species of listeria reported in different research article conducted in Ethiopia, *L. ivanovii* was the predominant bacterium isolated followed by *L. welshimeri* with prevalence of 29.7%) and 29.3%, respectively. The finding of this study is consistent with studies done elsewhere [64–67]. The highest prevalence of *L. monocytogenes* was reported in study conducted in Gondar town with prevalence of 32.7%. In this meta-analysis, *L. seeligeri* (2.8%) was the least bacterium isolated from food items of animal and plant origin.

In the present meta-analysis, raw beef was found to be the predominant food item contaminated with *Listeria* species, including *L. monocytogenes*. Out of 369 food items of animal and plant origin examined, around one third (29.3%) raw beef meat were contaminated with *Listeria* species, in which *L. monocytogenes* rated 11.1%. *L. innocua* was the predominant *Listeria* species isolated from this food item by account 61.1% of the total listeria species. This finding is similar with study conducted elsewhere [2, 68, 69]. The high contamination of the beef samples may be due to poor hygienic conditions during slaughtering, processing and selling. This indicates raw or undercooked beef meat was consumed traditionally throughout the country which aggravates the public health associated to *Listeria* species. Further processing and handling of meat also increases the risk of contamination with *Listeria* species [70, 71].

Similarly, we identified a significant level of contamination of raw milk with *Listeria* species (23.3%). *L. monocytogenes* the predominant bacterium isolated from raw milk with prevalence of 25.6% followed by *L. welshimeri, L. seeligeri* and *L. ivanovii* with prevalence ranged from 14 to 21%. This is consistent with studies conducted in different areas [60, 72, 73], where high contamination level of listeria species in milk and milk products identified. This high prevalence of listeria in milk might be due the tradition of mixing milk with water before selling to the consumer which increases the chances of contamination during dilution with water, poor personal hygiene or from contaminated environment and also poor milking practices [74].

In this meta-analysis, there was higher contamination of ice cream samples with *L. innocua, L. monocytogenes* and *L. ivanovii* with prevalence ranged from 10 to 47%. This might be due to the properties of the ice cream that facilitate a suitable condition for listerial growth and multiplication [58]. The increased contamination ice cream might be due the nature of this bacteria that can withstand a wide range of wide temperatures and other factor like, water contamination, and poor hygienic quality of food items sold in the country The consumption of animal product can change the nature of this bacteria which may drive emergence of new epidemiological patterns of disease [75, 76].

### Limitations of the study

The collected article for this study was limited to English language. Study method (most them were cross-sectional), which can affect the outcome variable by other confounding variables. Small sample size, which could affect the estimated pooled prevalence of *Listeria* species in different food items of animal and plant origin. Therefore, this meta-analysis represented only studies reported from limited study area, which may reflect under-representation due to the limited number of studies included.

## Conclusion

In this meta-analysis, there was high prevalence of *L. monocytogenes* and other *Listeria* species among different food items of animal and plant origin. In this study, *L. innocua* was predominantly isolated from cheese followed by *L.monocytogenes and L. murrayi. L. innocua* was the predominant isolated *Listeria* in raw beef, cream and egg, while *L. grayi was* the least isolate in raw beef and cream and *L. murrayi* was the least isolate in egg. Therefore, based on our findings, we recommend emphasis shall be given on health educations about cooking of animal product before consumption, improved food safety through the implementation of hygienic measures at all levels from production to consumption with particular emphasis on personal hygiene as well as, proper disposal of wastes including excreta in integration with the existing national health extension program are recommended.

## Data Availability

All data relevant to the study are included in the article.
